# Transvaginal Resection of a Bladder Leiomyoma Misdiagnosed with a Vaginal Mass: A Case Report and Literature Review

**DOI:** 10.1155/2015/981843

**Published:** 2015-11-26

**Authors:** Fu-Fen Yin, Ning Wang, You-Lin Wang, Xiao-Ning Bi, Xiao-Hui Xu, Yan-Kui Wang

**Affiliations:** ^1^Department of Obstetrics and Gynecology, Affiliated Hospital of Qingdao University, Qingdao 266000, China; ^2^Department of Urology, Shanghai Institute of Andrology, Renji Hospital, School of Medicine, Shanghai Jiao Tong University, Shanghai 200000, China; ^3^Department of Obstetrics and Gynecology, Peking Union Medical College Hospital, Chinese Academy of Medical Sciences and Peking Union Medical College, Beijing 100000, China; ^4^Department of Obstetrics and Gynecology, The Chinese People's Liberation Army 401 Hospital, Qingdao 266000, China

## Abstract

Bladder leiomyoma is a rare benign tumor and it could be easily misdiagnosed with many other pelvic diseases, especially obstetrical and gynecological diseases; abdominal, laparoscopic, and transurethral resection of bladder leiomyoma have been reported. Herein, we present a case of bladder leiomyoma misdiagnosed with a vaginal mass preoperatively; the mass was isolated, enucleated from the bladder neck, and removed transvaginally; to the best of our knowledge, this is the first case of intramural leiomyoma of bladder neck that has been enucleated transvaginally only without cystotomy.

## 1. Introduction

Bladder leiomyoma is a rare benign tumor; the symptoms depend on the location and size of the tumor; surgery is the standard treatment and the surgical approach depends on tumor size and localization at the bladder wall; this rare disease can be confused with many other pelvic diseases, especially obstetrical and gynecological diseases.

## 2. Case Report

**Figure 1:**
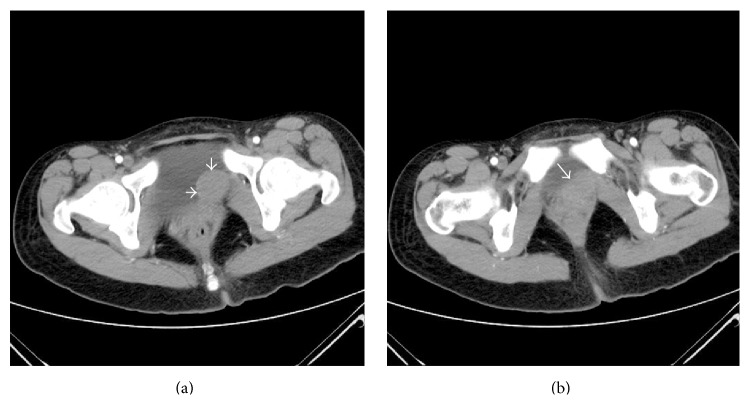
Contrast-enhanced computed tomography (CT). (a) shows a diameter of about 3 cm soft, mild enhancement mass at the left posterior wall of the bladder, protruding into the bladder locally. (b) shows the mass that was adjacent to bladder neck and vagina.

**Figure 2:**
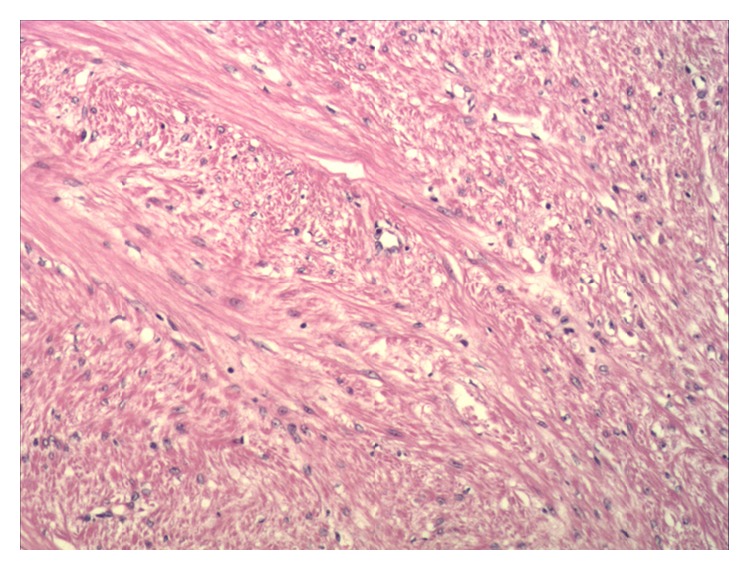
The pathological image of the bladder leiomyoma.

A 22-year-old woman was referred to our outpatient department. She complained of lower left abdominal pain without radiating to other parts of the body, which had occurred for the previous 2 months. She did not complain of any urinary or bowel symptoms such as urinary frequency, urgency, dysuria, or incontinence. She denied any significantly surgical or medical history. Bimanual pelvic examination detected a well circumscribed firm and slightly movable spherical lesion located in the left of the vaginal wall. Urinalysis was within normal limits. Ultrasonography (US) revealed a relatively irregular and heterogeneous mass in the left posterior wall of the bladder. Contrast-enhanced computed tomography (CT) scan demonstrated a 3.2 × 2.5 cm soft, mild enhancement mass near the left posterior wall of the bladder, protruding into the bladder locally (Figure 1). The cystoscopy found a hemispherical mass located in the left wall of the bladder close to the internal urethral orifice with intact urothelium, indicating that the mass was either external to the bladder or arising from it. The subsequent transurethral resection deep biopsy was normal. Based on clinical evaluation and all complementary findings, an equivocal diagnosis of pelvic mass could only be obtained. The tumor was resected transvaginally for diagnosis and treatment. The operative procedures were as follows: first, bilateral ureteral stent implantation was performed with cystoscope in order to protect the ureters; second, we separated the tumor through the vagina part and partly incised the bladder neck. We found that the mass was intramural of the bladder neck. Finally, we probed and removed the tumor completely without cutting through the whole bladder. The whole process was within simultaneously cystoscopic control. The resected mass had smooth surface and its measured diameter was proximately 3 cm. The cut surface of the tumor showed white laminated periphery. To avoid the possible postoperative complications of the surgery, such as cystocele, dysuria, urinary frequency, incontinence, or other urinary symptoms, we repaired pubocervical fascia carefully. The diagnosis of leiomyoma was confirmed on postoperative histopathology (Figure 2), of which the immunohistochemistry showed the following: Desmin (+), SMA (+), VIM (−), Dog-1 (−), CD117 (−), and CD34 (−), and positive rate of Ki-67 was 2%. The patient did not complain of any discomfort of urinary symptoms and was relieved of previous symptoms during the postoperative recovery. Over a 24-month follow-up period, it did not recur on US.

## 3. Discussion

Bladder leiomyoma is a rare benign tumor; the symptoms depend on the location and size of the tumor [1, 2]. A diagnosis can be made relying on the symptoms and complementary findings of US, CT, magnetic resonance imaging (MRI), and preoperative diagnostic biopsy [1, 3, 4]. Ultrasound is most commonly the first imaging step; MRI and CT may also be performed if this kind of tumor is suspected [5]. CT provides us with information about the size, position, and relationship between the tumor and bladder wall, but when comparing it with US and MRI its accuracy to define the relationship between the tumor and the bladder wall is inferior [6, 7]. MRI may better show the submucosal origin of the mass. However, MRI adds a new dimension to recognition and overall assessment of the tumor and cannot be relied on unfailingly to differentiate leiomyoma from leiomyosarcoma, because both may be enhanced after contrast media administration [8]. 50% of results of biopsy samples were false-negative and cystoscopic biopsy was an invasive procedure [6]. Therefore, all factors taken into consideration, US is considered the most useful and the first choice of imaging diagnostic tool for diagnosis of bladder leiomyoma [6, 9]. Nevertheless, we should combine US, CT, MRI, and cystoscope in the diagnosis of bladder leiomyoma. Though complementary tests add important data that suggest the benign nature of bladder leiomyoma, there is no test that allows us to differentiate leiomyoma from leiomyosarcoma; therefore, final diagnosis depends on histological study of the surgical piece [4, 6, 10, 11].

The treatment of bladder leiomyoma depends on the size, location, and involvement of urinary sphincter or ureter orifices and the patient's preference [12]. For small and endovesical tumors, transurethral resection (TUR) is recommended, whereas segmental resection is recommended for bigger, endovesical, intramural, or extravesical tumors [13, 14]. Operative measures of an open surgery, laparoscopy, and transurethral resection have been reported [2, 13, 15]. Herein, to our knowledge, this is the first case of an intramural bladder leiomyoma managed successfully by a transvaginal enucleation. Though transvaginal resection (TVR) is recommended for extravesical tumors adjacent to vagina, the uncertain types which are assessed anatomically near the vagina may also be managed by TVR. Postoperative recovery of the patient was uneventful and the patient was relieved of previous symptoms. No recurrence was found on US over a 24-month follow-up period.

Most bladder leiomyoma shows typical benign tumor characteristics and can achieve a definite diagnosis through preoperative biopsy. But some can be obscured with many other pelvic diseases. Lower urinary tract leiomyomata especially should always be considered in the differential diagnosis of an anterior vaginal mass[15]. We cannot achieve a definite diagnosis based on the existing symptoms and the complementary findings [4, 10]. The definite diagnosis translates to the optimal and minimally invasive therapeutic measures. In order to improve the differential diagnosis, we have reviewed cases of bladder leiomyoma without a definite diagnosis preoperatively. All of these cases have been blurred with other pelvic diseases, especially urogenital diseases. Bladder leiomyomata are rare differential diagnoses to other pelvic diseases including uterine mass, ovarian tumors, acquired dysmenorrhoea, female pseudoprostate, stress incontinence, and impotence [1, 3, 12, 16–24]. Bladder leiomyoma and uterine myomas exhibit similar characteristics on US, CT, and MRI [1, 3, 12, 16, 17]. A hysterectomized woman, presented with hematuria, abdominal pain, and palpable pelvic mass, with a normal cystoscopy, was suspected of an ovarian tumor, finally demonstrating a multiple bladder leiomyoma [18]. Banerjee et al. presented a case where a giant bladder leiomyoma measuring 21 × 17 × 16 cm was mistaken to be an ovarian mass [19]. Bladder leiomyoma can also be responsible for acquired dysmenorrhoea in some women, leading to gynecological assessment [20]. Lee et al. reported one female patient with leiomyoma of bladder who presented with female pseudoprostate and right hydronephrosis, revealing that bladder leiomyoma might be one of the differential diagnoses with CT imaging findings of female pseudoprostate [21]. Bladder leiomyoma should also be considered as a rare cause of urinary stress incontinence and impotence [22–24]. Urinary bladder leiomyoma associated with a medical history of pulmonary lymphangioleiomyomatosis has been reported; thus, bladder leiomyoma should be considered in the differential diagnosis of a patient who presents with a genitourinary mass and a history of LAM [25].

There are many proposed etiologies for leiomyoma, including chromosomal anomalies, hormonal changes, infection of bladder smooth muscle, perivascular inflammations, and dysontogenesis [26]. Many findings have shown that bladder leiomyoma occurs more frequently in women than in men [12, 27–29], indicating that gender differences exist on the bladder leiomyoma occurrence. It is proposed that women in their third, fourth, fifth, and sixth decades of life most commonly encounter leiomyomas [26]. The bladder leiomyoma during pregnancy increases more obviously in size and reoccurs more frequently [27, 30–33]. Cases of bladder leiomyoma with ovarian steroid hormone receptors have been reported [27, 33]. All of these results reveal that bladder leiomyoma may be dependent on hormonal changes as uterine leiomyoma. By exploring roles of steroid hormone on this disease, we can assess the bladder leiomyoma with other perspectives; it may facilitate proper measures of the prevention, diagnosis, and therapy for this disease. The use of gonadotropin-releasing hormone may be another choice for the treatment of bladder leiomyoma as in uterine leiomyoma. GnRH analogues can be effective for uterine leiomyoma, because it can influence hypothalamus-hypophysis-gonadal axis and play a role as medical oophorectomy [34]. GnRH has been predicted to be effective for the bladder leiomyoma as well as uterine one because bladder leiomyoma was also reported to be associated with hormonal changes as uterine leiomyoma. However, we propose that GnRH treatment is not applicable for males because, for males, estrogen is mainly from adrenals but GnRH mainly plays a role in hypothalamus-hypophysis-gonadal axis. So gonadotropin-releasing hormone may be specially fit for women who still have ovarian function but are approaching menopause and for women who have undergone myomectomy but wish to prevent recurrence or to arrest the development of an additional leiomyoma.

In conclusion, though TVR is recommended for extravesical tumors, we firstly present a transvaginal resection of an intravesical leiomyoma. TVR is another feasible, minimally invasive surgical approach for intravesical leiomyoma of bladder neck. Bladder leiomyoma can be obscured with many other pelvic diseases; it should be considered as a rare cause of uterine mass, ovarian tumors, acquired dysmenorrhoea, female pseudoprostate, stress incontinence, and even impotence. Steroid hormone is correlated with bladder leiomyoma; gonadotropin-releasing hormone may be a new choice for the prevention, diagnosis, and treatment of bladder leiomyoma.
